# Dealing With Clozapine-Induced Sialorrhea

**DOI:** 10.1192/j.eurpsy.2022.1871

**Published:** 2022-09-01

**Authors:** J. Sá Couto, B. Da Luz, M. Pão Trigo, J. Rodrigues, T. Ventura Gil

**Affiliations:** Centro Hospitalar Universitário do Algarve, Departamento De Psiquiatria E Saúde Mental, Unidade De Faro, Faro, Portugal

**Keywords:** Sialorrhea, schizophrénia, clozapine

## Abstract

**Introduction:**

Clozapine is the first atypical antipsychotic. It is used in refractory schizophrenia. It has a heavy side effect burden, including weight gain, dizziness, blurred vision, and sialorrhea. Not only is sialorrhea bothersome, but it can also have with serious consequences, such us aspiration pneumonia, neutropenia, agranulocytosis, myocarditis, and may be responsible for low self-esteem, leading to low treatment compliance and discontinuation.

**Objectives:**

Identifying the mechanism behind clozapine-induced sialorrhea. Finding how frequent clozapine-induced sialorrhea is compared to other antipsychotics. Finding effective ways to prevent clozapine-induced sialorrhea.

**Methods:**

*PubMed* database search, with *“clozapine sialorrhea”* keyword expression. 12 Articles published in the last ten years were selected among the 112 best matches. Reference lists of articles were reviewed to identify additional articles.

**Results:**

Clozapine is a muscarinic M1-5 receptor antagonist, explaining its anticholinergic effects. Due to its strong anticholinergic action, sialorrhea is a paradoxical side effect. To prevent it, several drugs can be used, such us scopolamine, pirenzepine, sublingual atropine solutions, clonidine, botulinum neurotoxin, and others. Sialorrhea was relatively more frequently reported in clozapine (1.1%) compared with other antipsychotics (0.31%). Mubaslat and Lambert (2020) found that drops of atropine reduce the rate of saliva secretion significantly better than placebo. Uzun, et al. (2019) observed the adjunction of N‐acetylcysteine allowed a significant decrease of the severity of sialorrhea and was well tolerated.

**Conclusions:**

Although effective in refractory schizophrenia, clozapine side effects, namely sialorrhea, can be bothersome and may affect treatment adherence. Fortunately, we have tools at our disposal to help patients better handle it.

**Disclosure:**

No significant relationships.

